# An integrated model for developing research skills in an undergraduate medical curriculum: appraisal of an approach using student selected components

**DOI:** 10.1007/s40037-013-0079-7

**Published:** 2013-09-14

**Authors:** Simon C. Riley, Jeremy Morton, David C. Ray, David G. Swann, Donald J. Davidson

**Affiliations:** 1 Centre for Medical Education, Chancellor’s Building, University of Edinburgh, Edinburgh, UK; 2 Anaesthesia and Critical Care, University of Edinburgh, Edinburgh, UK; 3 MRC Centre for Inflammation Research, University of Edinburgh, Edinburgh, UK; 4 MRC Centre for Reproductive Health, Queen’s Medical Research Institute, 47 Little France Crescent, Edinburgh, EH16 4TJ Scotland, UK

**Keywords:** Learner-centred, Experiential learning, Research skills, Research competencies, Curriculum development, Learning outcomes, Student choice, Student selected components

## Abstract

Student selected components (SSCs), at that time termed special study modules, were arguably the most innovative element in Tomorrow’s Doctors (1993), the document from the General Medical Council that initiated the modernization of medical curricula in the UK. SSCs were proposed to make up one-third of the medical curriculum and provide students with choice, whilst allowing individual schools autonomy in how SSCs were utilized. In response, at the University of Edinburgh the undergraduate medical curriculum provides an integrated and sequential development and assessment of research skill learning outcomes, for all students in the SSC programme. The curriculum contains SSCs which provide choice to students in all 5 years. There are four substantial timetabled SSCs where students develop research skills in a topic and speciality of their choice. These SSCs are fully integrated and mapped with core learning outcomes and assessment, particularly with the ‘Evidence-Based Medicine and Research’ programme theme. These research skills are developed incrementally and applied fully in a research project in the fourth year. One-third of students also perform an optional intercalated one-year honours programme between years 2 and 3, usually across a wide range of honours schools at the biomedical science interface. Student feedback is insightful and demonstrates perceived attainment of research competencies. The establishment of these competencies is discussed in the context of enabling junior graduate doctors to be effective and confident at utilizing their research skills to effectively practice evidence-based medicine. This includes examining their own practice through clinical audit, developing an insight into the complexity of the evidence base and uncertainty, and also gaining a view into a career as a clinical academic.

## Introduction

In the first edition of Tomorrow’s Doctors in 1993 [1], the General Medical Council (GMC) prompted a thorough overhaul of UK undergraduate medical curricula, to reduce factual content that was perceived to be unnecessary, and refocus on other professional skills. Probably the most radical change was provision of extensive student choice, through what were originally termed special study modules (SSMs). ‘As a general guide we would expect approximately a third of the total undergraduate programme to be devoted to [SSMs] … that at all stages of the course students will be engaged in some work outside the core syllabus…’. In 2003, these SSMs were subsequently renamed as student selected components (SSCs) [1].

A short history and context is helpful to show how this GMC prompt on SSMs/SSCs was implemented into the medical curriculum at the University of Edinburgh. Our medical school senior curriculum management team had the vision to recognize not only the challenges, but also the opportunities presented by this radical curriculum transformation for developing students’ research skills, and their professionalism. Now the medical curriculum has a fully integrated research theme that runs through the whole programme curriculum and timetable, delivered within the SSCs, where students have choice in their field or speciality of study in medicine. These SSCs integrate with the evidence-based medicine elements in the curriculum to develop research-based competencies. As a research-rich medical school and institution, it was perceived that some educational opportunities were under-utilized, including the engagement of more research active faculty in undergraduate teaching. The educational benefits of undergraduates performing research was perhaps not well defined or lacked specific evidence at that time. However, there was already a strong intercalated honours programme which about 30 % of students undertook, with anecdotally the perception that these students make substantial educational gains. At the same time, our senior curriculum management team were playing a role in the Scottish Doctor [2] project, defining the Scottish medical curricula in a taxonomy and framework of learning objectives. So throughout our curriculum development, this type of integrated framework has been implemented, established and accepted. Nevertheless, it should also be stressed that implementation was a challenge that needed careful management [3].

The current medical curriculum at the University of Edinburgh dedicates 20 % of curriculum time to SSCs, spread throughout the 5 years. Of this SSC timetable, three-quarters are dedicated to students engaging in research projects, and the other quarter to non-research SSCs. Although this amounts to much less than the one-third that was originally required in the 1993 edition of Tomorrow’s Doctors [1], this has been robustly defended as appropriate pointing to the delivery of authentic student choice, quality of experience, and integration of learning. At some other medical schools in the UK, their SSC programmes have been perceived to be somewhat compromised, by badging as SSCs core clinical attachments that lack real autonomy and choice, as detailed in the Quality Assurance of Basic Medical Education reports arising from GMC visits to review schools (QABME) [4]. This view that one-third was too difficult to deliver without compromising the ethos of genuine student choice was recognized in time by the GMC, and the third and current iteration of Tomorrow’s Doctors of 2009 [1] states a >10 % timetable requirement for SSCs.

Expert performance is a complex activity, requiring a deep understanding and knowledge, and acceptance of uncertainty and risk [5]. Problem solving is key, where incomplete and perhaps conflicting information needs to be synthesized and processed effectively. In our undergraduate medicine curriculum, we aim to establish a learner-centred experiential approach, which is educationally beneficial [6, 7] to develop deep learning with higher order intellectual skills and attributes, and improve clinical diagnostic skills and pattern recognition from within an integrated curriculum [8, 9]. Research skills can be superficially offered as a series of lectures, workshops and small-group work, applying a range of techniques. However, in our own curriculum we show it is feasible for students to perform research studies themselves to attain higher level research skills with experiential student learning, as demonstrated across a range of other subject areas in tertiary education [10–12]. Medical education should take an outward looking approach at this research-teaching nexus [13, 14]. The limitations should also be recognized with the short duration of any projects, variability in experience, and perceived conflict with other parallel learning activities.

It is important that the term ‘research’ in the contexts of both our own curriculum, and more generally in curricular design in medicine should be defined broadly as obtaining new knowledge [15–17]. It should include the extensive range of competencies and skills needed to define the knowledge gap, find and access the background information, critically assimilate and appraise the existing primary research literature, formulate a scientific question, design and utilize appropriate methods to address the question, collect and analyze data and then critically review and present the findings in context. The definition here is taken to include clinical audit and service evaluation, which aims to establish if best practice is being followed, and identifies where there is a paucity of evidence. These types of study do involve obtaining new knowledge [15].

Evidence-based medicine has become established as an essential element in medical practice. Now the medical practice in health boards, hospitals, and clinical units is (perhaps to the granularity of individual clinicians) audited and compared with national guidelines or success rates. Clinicians have to engage with service evaluation and audit of their local practice. This forms an important driver to fully establish research skills in all medical curricula.

## Integrating research into the curriculum: developing, defining and mapping the evidence-based medicine and research elements

The Edinburgh medical curriculum has undergone extensive mapping to establish what learning outcomes are initiated, developed and attained, then blueprinted to the assessment to establish intermediate and final checkpoints. It is also mapped and aligns closely to Tomorrow’s Doctors [1]. The most recent development is to make this mapping available to students, faculty and educators to facilitate learning, assessment and curriculum management. This mapping and blueprinting exercise has required significant management and resources, and it is clear from QABME reports [4] that it has proved a similar challenge in many UK medical schools. The main learning outcomes and competencies pertaining to research that define the Edinburgh curriculum are mapped to Tomorrow’s Doctors [1], as detailed in Table 1. The main difference is they are more clearly defined and extend beyond Tomorrow’s Doctors, to performing simple research studies.

**Table 1:** Alignment of the “research” learning outcomes the Edinburgh Medical Curriculum SSC programme with Tomorrow’s Doctors [1]

	Tomorrow’s Doctors (GMC [1])	Edinburgh MBChB learning outcomes
12	*Apply scientific method and approaches to medical research*	*PROGRAMME THEME: Evidence Based Medicine and Research (EBM&R)*
		The Edinburgh medical graduate will be able to: Use the best available medical evidence, found through a systematic search and appraisal of the relevant information sources, to inform their clinical decisions; and develop new knowledge or personal understanding through the application of basic research methods and skills
12(b)	Formulate simple relevant research questions in biomedical science, psychological science or population science, and design appropriate studies or experiments to address the questions	Formulate straightforward, relevant research questions for literature reviews and design appropriate studies or experiments to address the questions
		Perform simple medically relevant research and audit studies to collect and analyze data
19(d)	Access information sources	Identify and interrogate appropriate sources of information, including bibliographic databases
12(a)	Critically appraise the results of relevant diagnostic, prognostic and treatment trials and other qualitative and quantitative studies as reported in the medical and scientific literature	Critically appraise relevant diagnostic, prognostic and treatment trials and other sources of information including qualitative and quantitative studies
12(c)	Apply findings from the literature to answer questions raised by specific clinical problems	Apply findings from the literature to answer questions raised by specific clinical problems, relating to patient care, health promotion and giving advice and information to patients
19(d)	And use information in relation to patient care, health promotion, giving advice and information to patients, and research and education	Apply findings from own research and literature studies for clinical practice, further research and education
19	*Use information effectively in a medical context*	*PROGRAMME THEME: Medical Informatics (MI)*
		The Edinburgh medical graduate will be able to: Use computers, computing, information and information technology effectively in a medical context
19(b)	Make effective use of computers and other information systems, including storing and retrieving information	Make effective use of computers and other information systems, including storing and retrieving information
		Make appropriate and safe use of electronic communications
		Make appropriate and effective use of electronic health records
19(c)	Keep to the requirements of confidentiality and data protection legislation and codes of practice in all dealings with information	Keep to the requirements of confidentiality and data protection legislation and codes of practice in all dealings with information including audit and research
		Apply the principles, method and knowledge of informed consent protocols when handling and/or transferring patient data
19(e)	Apply the principles, method and knowledge of health informatics to medical practice	Apply the principles, method and knowledge of health informatics to medical practice
	*The doctor as a professional*	
20	*The graduate will be able to behave according to ethical and legal principles. The graduate will be able to:*	*PROGRAMME THEME: Medical Ethics, Legal and Professional Responsibilities (MELPR)*
		The Edinburgh medical graduate will be able to: Practise medicine safely, within an ethical framework, with insight and compassion, according to the legal requirements and professional expectations of medical practice in the UK
12(d)	Understand the ethical and governance issues involved in medical research	Demonstrate an understanding of the ethics and regulation of research with humans and animals in biomedical research, clinical research and audit, and of critical reflection on the interests of individuals and society

In the Edinburgh curriculum, learning outcomes run vertically through the curriculum in ‘Programme Themes’. All the learning outcomes are owned by these themes, with the modules and specialities teaching them in their context. Similarly, the SSC is a timetabling scaffold on which to build learning opportunities, although they are characterized by student choice. The majority of learning outcomes delivered by SSCs are within the ‘Evidence Based Medicine and Research’ and the ‘Personal and Professional Development’ programme themes, although ‘Medical Informatics, ‘Medical Ethics, Legal and Professional Responsibilities’, and ‘Clinical Communication’ also feature significantly. With the clear statement in Tomorrow’s Doctors [1] that ‘depth of study’ amounts to a competency in its own right, all learning outcomes achieved within the SSC domain can be viewed as ‘core’ [18]. For each SSC, every student follows the same format and assessment process, permitting confidence that all students consistently attain all the learning outcomes. The core competencies can be delivered in any clinical or biomedical setting, and with a little consideration can even be attained in fields outside medicine, facilitating genuine student choice [19, 20].

## Description of the SSC programme

In years 1 and 2 there are approximately 220 students enrolled. This increases to around 260 with a group of 40 entrants joining directly at the beginning of year 3. Students take SSC1 in the second semester of year 1. Students individually select from a wide range of simple research/audit projects, to form groups of 7–8 students. Projects are usually offered in hospital disciplines, which provides some diversity for students as their clinical teaching is based in primary care at this time. These projects are frequently very simple service evaluations of the patient journey in a hospital clinic. The student group collect and critically review their data, and present it as a poster in the context of the existing literature for assessment. They also provide insight into their group dynamic and teamwork, offering anonymous individual constructive peer feedback using an online system within the virtual learning environment (VLE), the Edinburgh Electronic Medical Curriculum [21].

An SSC is performed in each of the two semesters in year 2. For SSC2a in the first semester, students use a rankings-based online chooser system to select a topic from an extensive list of clinical and/or scientific issues, and groups of 7–8 are formed according to their selections. Following introductory taught sessions on literature searching and critical appraisal, each group, guided by a tutor, researches and critically appraises the literature on their chosen topic. This involves discussions and presentations in a series of tutorials, leading to the presentation of a 6,000 word wiki report, which includes a critical appraisal of a key paper. The wiki requires individual students and the groups to demonstrate depth of understanding in addressing these well-focussed research questions. The students also apply the critical appraisal training they receive simultaneously in teaching in public health and statistics. Assessment is performed by a small group of faculty assessors for consistency. For SSC2b in the second semester of year 2, all project topics are self-proposed by groups of 5–8 students themselves [22, 23]. The group has to find their own tutor, although they can seek advice from the faculty lead. Students can choose any topic, including outside medicine, although most topics are within the field, and unlike SSC2a projects which are entirely literature-based, may take the form of a simple project with some data collection and analysis (e.g. clinical audit or survey). The output is again a wiki, in the same format as SSC2a. These wikis are made available externally as a view into the medical curriculum through the VLE, for example for potential applicants to the programme [24]. The main learning outcomes, although perhaps less obvious to the students at the time, derive from the establishment of an autonomous self-directed learning environment, including the development of team working, organizational and self-motivational skills. Both SSC2a and SSC2b use the same online peer feedback system as SSC1.

In year 4, all students self-propose an 8-week research project, in an area of medicine of their choice. This is embedded in a 14-week block with other clinical teaching. It represents an additional challenge and an important professional skill for students to effectively manage their time and project. The majority (approximately 60 %) of these research projects are some form of clinical audit or service evaluation, most frequently as part of an audit cycle examining local practice. However, some audit-type projects with a more ambitious study design may utilize a national or international database, which can be viewed as going somewhat beyond audit and contributing to the evidence base. The remaining projects usually link a student into an established clinical research group, or involve some sort of literature study, perhaps a systematic review. The timing of this substantial project in year 4 is considered optimal, allowing students to both build on their research competencies, and also for career exploration, as it is just prior to the job application process in the UK. Any later, and students are more focussed on gaining clinical skills to be competent for their initial job post-graduation [25].

For completeness, to describe the entire SSC programme, students undertake two more SSCs. For SSC3 in year 3, they perform a short SSC where individual students self-propose and arrange 4–6 afternoon sessions shadowing a health care professional who is not a doctor. This focuses on analyzing and reflecting on the wider clinical team and multi-professional working. In year 5, students undertake the widely acknowledged and understood ‘clinical elective’ over 8 weeks [26]. This is based away from the home institution, most frequently abroad in a resource poor, emerging or developed country, depending upon the professional and personal aims and learning outcomes the student establishes for themselves in discussion with their faculty Personal Tutor.

Students do have another major element of choice to undertake research in our medicine programme, although it lies outside SSCs. They can choose to participate in a 1-year BMedSci intercalated honours degree, between the second and third year. This opportunity is currently limited to 40 % of students in the year, with academic ranking used to ascertain final acceptance, although in practice, most students who wish to take the opportunity can. Students select from a wide range of 20 honours programmes, predominantly within biomedicine and biosciences, where medical students integrate with science students for the year. Honours programme choices, in order of popularity in the last 2 years, have been neuroscience, psychology, reproductive biology, sports science, physiology, international public health policy, pharmacology, immunology and infectious diseases. Students are also permitted to undertake an honours programme at another institution, if the topic is not offered by the home institution, and up to 5 % of students take this. Participation in these honours programmes allows students to develop a much greater insight and critical view into the underlying science of their chosen field, as well as insight into research and academic medicine [27]. All honours students undertake a substantial project, often laboratory-based, during semester 2. Faculty sometimes indicate a preference for students with this intercalated degree when selecting a student to work with them on the SSC4. They perceive these students bring a range of skills and a professional maturity that enhances the project [28–30].

## Assessment of research-based SSCs

Assessment of research-orientated SSCs represents a challenge [31]. The competencies and learning outcomes need to be clearly defined. Short research projects contain a significant risk that they may not produce any valid results for a wide range of reasons, many of which may be largely outside the control of a student, who likely compound these as they will be inexperienced in problem solving. In part, this stems from the lack of time to rectify any problems that arise, and differs from a higher degree such as a PhD, where any problems would be expected to be adequately addressed. SSCs provide an environment in which students can make a mistake as they are learning the research process. The assessment must be able to respond impartially and not be reliant upon a narrow interpretation of the success of a project which is solely based on the validity of the results. This view of assessment differs from many other forms of assessment across medicine, where core knowledge may be essential to establish competency. As long as students can demonstrate that they have learned from any mistakes or problems, and have offered a critical view of their findings, and insight into other approaches for the future, they should be able to pass the assessment.

All students, either as a group or individually, are required to provide some sort of reflection on the experience, which can be revealing and insightful. Questions an SSC examination board asks itself may include: ‘Has the student reflected and gained insight? Would they be able to act more appropriately to challenges in the future? What is to be gained by this student having to redo some, or all, of this SSC?’ Learning from mistakes is an essential skill, and in this way competency can be viewed by how the student has engaged with the process and their learning. All students develop an extensive personal e-portfolio throughout the course. It contains all their SSC reports, together with a linked ethics review and reflective practice task. The e-portfolio is also made up of a wide range of other elements from elsewhere in the core curriculum, including case reports which form an important part of assessment.

A range of issues needs to be considered in the validity and robustness of project marking. There are hawks and doves in supervisors who perform the marking [32]. There may also be halo and leniency effects if the supervisor is marking their own student’s project, although the supervisor is best placed to offer detailed and insightful feedback [33]. A supervisor may get to know the strengths and weaknesses of a student very well over an extended period, and this constructive criticism should be valued highly. Equally, it can be viewed that these variations in marks reflect genuine differences in opinion. Single marks for group projects are perceived by individuals as being unfair, and can raise levels of complaints from a small number of students that is out of proportion to the contribution of the mark overall. Students need to develop and demonstrate some resilience. It is invaluable for students to recognize that teamwork is more important than individual effort in achieving common goals. This prepares them for working in clinical teams. To try and best address these complex assessment issues, the marking schemes and assessment schemes have been carefully designed, and students receive marks and written feedback from their tutors and supervisors, independent markers, and also their peers. Students are also tasked with providing their e-portfolio material to their personal faculty academic tutors for discussion and decoding, as evidence of their reflective practice and progression. When appropriate, faculty SSC course leads will contact a student’s personal tutor to address these questions of perceived unfairness.

## Provision of resources

The SSC programme has substantial resources that students can access. Each individual SSC has a faculty lead, with a Director of SSCs who oversees the whole programme. There is a dedicated SSC administrator who provides support to the faculty leads of each of the SSCs, to students, and to the examination boards on assessment.

From first year, all these research SSCs have to undertake an ethical and governance screening review. This is an essential e-portfolio item for all students, and integrates with the teaching in the ‘Medical Ethics, Legal and Professional Responsibilities’ programme theme. Ethical review can be perceived as a potential impedance to students performing research [34]. In these SSCs students are performing a study with an often overriding educational component. Furthermore, it is most frequently not a hypothesis-led research study, being better defined as a clinical audit or service evaluation. These types of study are well defined by the UK National Research Ethics Service [35] and do not need research ethics approval. The University of Edinburgh has had a long-term agreement with the local research ethics committee to facilitate guidance and a preliminary screening process, to establish if further ethical approval and a full submission is required to the research ethics committee. With their supervisors, students work through a simple questionnaire, with clear guidance in the course study guide and VLE, and facility to contact a faculty medical ethicist if required.

A dedicated medical statistician is available and complements skills developed earlier by students delivered by the public health specialists within the integrated curriculum ‘Evidence-based medicine and research’ theme. This statistician supports student learning in applied statistics, offering extensive online learning resources, and individual consultations [36]. There are workshops offered by medical librarians which are integrated as part of the ‘medical informatics’ programme theme, together with more advanced workshops, for instance on systematic literature searching if required. The librarians are also available for individual consultations.

Funding student research projects is a further challenge. These generic resources and support for ethical review, applied statistics and literature review are important, but individual projects may still require significant further resources or funding. For instance, a laboratory project can only be expected to be made available by a well-funded research group with external funding. Nevertheless, other audit type studies may require much less or minimal funding, with student participation removing salary costs. Monies allocated to teaching may be channelled to support individual projects. Other innovative models exist to provide project funding, including applying a tariff to each SSC, then giving students a fixed budget to fund their SSC programme, which they have to manage themselves [4].

## Self-proposal of SSCs by students from early in the curriculum

All students self-propose their SSCs from the middle of second year. Self-proposal requires students to take the initiative, to contact potential supervisors and generate a project. Taking the initiative to arrange their project forms an important learning outcome in itself [37]. Students receive assistance in this self-proposal process, with extensive guidance, information on previous projects and supervisors with their contact details. Furthermore, the faculty course lead provides input to an online notice board, where faculty supervisors can indicate their willingness to act as a point of contact in their medical speciality, or offer a potential idea for a student project. At no point are students allocated to a project or supervisor. There is something of a competitive edge to secure a project and faculty supervisor, and a potential supervisor approached by multiple students can elect to select a student based on an informal interview process.

## Student and faculty aspirations: alignment to ensure sustainability

Ensuring sustainability of the SSC programme year-on-year is vital. Recognizing the alignment of both faculty and student aspirations is an important element to achieve this [13]. Faculty members are engaged and they recognize the benefits of working with relatively ‘time-rich’ students to assist them with clinical audit, service evaluation, surveillance and patient safety studies, which they are tasked with as part of their own practice—the projects performed are considered useful. Faculty also keep on returning in following years with more projects, which may be entirely new, or as a follow-up of a previous project, frequently a student-led audit. These types of project also form an opportunity to engage a wider pool of clinicians with the curriculum and medical education, contributing to this sustainability. This also spreads the teaching load so it is not too onerous, particularly the assessment process, which should be focussed and recognized as offering valuable feedback. There is often an altruistic element, with faculty looking to attract junior staff into their own speciality. These research-orientated SSCs in years 1, 2 and 4 engage with over 250 members of faculty each year, who contribute through supervision, organization and assessment. This illustrates that the alignment and learning environment is effective, and that a significant teaching load is being spread. Faculty regularly indicate to the course leads they are considering dropping out of supervision for a year. This would occur if projects were collated by the course leads. However, an enthusiastic student who has to self-propose can frequently re-invigorate that jaded member of faculty with their independent approach.

For students, there are a complex series of motivations that are involved in decision-making as to the choice of project [23, 38]. Self-selection of a medical speciality and project often reflects a possible career interest, so they are motivated, enthusiastic, and want to impress their faculty supervisor who may become a potential mentor, referee or colleague. Their motivations have been categorized in a research-rich SSC programme and not unexpectedly perhaps shown to change with the year of study, with adjustments in student’s agenda, insight and aims with progression [38]. Even early in the programme some sort of research exposure generates an interest to look to further opportunities [39]. We and others recognize the importance of integration into the research community [15]. Students often, unrealistically, see an opportunity to gain a publication that can further enhance their curriculum vitae [40]. Publications (defined as peer-reviewed journal articles and reviews, and as abstracts presented at national and international conferences, but not at student meetings) arise from approximately 10 % of all projects performed from SSC4 projects. This seems to be comparable with publication rates at some other institutions with similar types of programmes [41, 42]. However, the number of local clinical audit or service evaluation studies that have influenced local health care delivery will be higher, and this needs to be better defined.

## Is the programme effective? The student view

As part of the quality assurance process, feedback is collected from students across all years and courses within the medicine programme, using an online questionnaire. To reduce questionnaire fatigue, students are selectively sampled, focussing on individual courses if there are perceived problems, or new innovations being implemented. This feedback has been useful in identifying and then responding to issues in different parts of the SSC programme, including improving the online peer feedback system, assessment, feedback provision and resource provision. When viewed holistically across the whole SSC programme, it is useful in identifying good practice and integration (Table 2). It highlights that students recognize they incrementally continue to gain a greater depth of understanding and depth of study. Further, they continue to develop their research skills in critical appraisal, informatics, statistics and data handling, and a range of professionalisms, including teamwork, time and workload management, understanding of medical ethics, and a range of communication skills. Overall, they indicate that the tasks presented by their SSC become increasingly challenging. Almost all students rate very highly the final research SSC in year 4, where they perform a project with a senior member of staff.

**Table 2:** Compiled students responses from online quality assurance questionnaires, from 2008 to 2012

	Strongly agree/agree (%)	Neither (%)	Disagree/strongly disagree (%)
I have been able to gain a greater depth of understanding in this topic/project/field than is usual in other parts of the course			
Year 1—SSC1	88.0	8.0	4.0
Year 2—SSC2a	92.6	3.8	3.5
Year 4—SSC4	94.9	4.0	1.0
I have further developed my critical analysis skills during this SSC			
Year 1—SSC1	73.6	18.3	8.1
Year 2—SSC2a	89.7	4.8	5.5
Year 4—SSC4	92.6	5.1	2.3
This SSC has enabled me to further develop my statistical/data analysis skills			
Year 1—SSC1	47.7	28.4	23.9
Question not asked as no data handling in this year 2 SSC			
Year 4—SSC4	88.5	9.2	2.3
This SSC has enabled me to further develop my informatics/IT skills			
Year 1—SSC1	48.3	25.4	26.1
Year 2—SSC2a	71.0	16.8	12.3
Year 4—SSC4	91.5	6.8	1.7
I have further developed my skills and awareness for working in a team			
Year 1—SSC1	92.0	6.3	1.7
Year 2—SSC2a	89.4	7.1	3.5
Year 4—SSC4	74.1	15.5	10.3
The SSC1 has enabled me to further develop my independent management skills			
Year 1—SSC1	82.2	12.5	5.2
Year 2—SSC2a	80.1	12.8	7.1
Year 4—SSC4	96.6	2.8	0.5
I have gained further understanding of some of the ethical issues underpinning medicine during this SSC			
Year 1—SSC1	58.7	26.6	14.7
Year 2—SSC2a	44.3	25.6	30.5
Year 4—SSC4	74.4	17.2	8.3
I have further developed my presentation skills (this includes written, oral and visual formats)			
Year 1—SSC1	84.5	12.1	3.3
Year 2—SSC2a	74.4	14.7	10.9
Year 4—SSC4	84.5	8.6	6.9
This SSC was challenging			
Year 1—SSC1	75.1	15.7	9.1
Year 2—SSC2a	76.3	17.9	5.8
Year 4—SSC4	94.9	3.4	1.7

	Excellent/good	Average/reasonable	Poor/awful
Overall I would rate this SSC as			
Year 1—SSC1	75.9	18.9	5.1
Year 2—SSC2a	67.7	25.9	6.4
Year 4—SSC4	90.3	8.5	1.1

To reduce questionnaire overload, not all students were sampled, although there was remarkable consistency throughout between different years for each question in each SSC. Response rates are 287 of 469 (61.2 %), 312 of 479 (64.7 %), and 177 of 322 (55.0 %), for years 1, 2 and 4, respectively

Perhaps the most insightful feedback commentaries from students are in the qualitative free text (Table 3). These are almost overwhelmingly positive, with students recognizing the opportunity, reflecting on the array of skills used and attained, and appreciating the insight gained from working with a member of faculty. Negative comments tend to reflect either a significant problem arising with the ability to deliver the planned project, which is outside the control of the student and supervisor, or discontent about the assessment process from students who have not grasped the essential teamwork component and feel that the group mark awarded does not sufficiently reflect their personal contribution. Nevertheless, these scenarios are recognized in the stated learning outcomes, and in the assessment process, and most students can appreciate this.

**Table 3:** Feedback comments on their research SSCs

**Comment on the best aspects of your SSC**
‘Being able to work as part of a group; this was much more enjoyable and less isolating than ‘self-study’; the opportunity to actually produce a piece of work (as opposed to simply memorizing facts), that would be submitted and graded’ (SSC2a, 2010)
‘It gave me the push I needed to really apply critical skills when reading articles and reviews. It was liberating in the case that I got to write what I thought for once’ (SSC2a, 2010)
‘SSC4 allowed me the opportunity to explore my chosen area of medicine further. I gained much in the way of organization and data management skills, and feel competent now in statistical analyses. My tutor was very supportive and the whole process was very rewarding’ (SSC4, 2011)
‘I loved the opportunity to study something I really enjoyed, get a lot of clinical experience and have the experience of doing clinical research in a supported environment’ (SSC4, 2011)
‘Having to undertake a project but take a lot more responsibility for appropriate self-learning, organization and work’ (SSC4, 2011)
‘Learning new skills—systematic review, data analysis, graphs, statistical techniques. My supervisor was excellent—I really enjoyed working with her. Being able to manage my own time and set my own goals’ (SSC4, 2011)
‘Having the opportunity to explore an area you are interested in, and contribute something useful!’ (SSC4, 2010)
‘I really enjoyed the independence to investigate and area of interest. I also felt getting to be part of a new team and meeting people in a field I’m interested in invaluable’ (SSC4, 2010)
**Comment on why you selected this topic area/subject/speciality for your SSC**
‘I am keen to work in the speciality that I chose for my SSC and this gave me a good opportunity to learn more about the topic area and the challenges of working in a busy department’ (SSC4, 2011)
‘I wanted to do an SSC4 in a field to which medical students receive little clinical exposure, to broaden my learning experience’ (SSC4, 2010)
‘I had a particular interest in the area of my project before starting, and thought this would be a good way of finding out more about it and gaining an insight into the speciality’ (SSC4, 2009)
Reflect and comment on any changes that you should have implemented to improve the experience and outcomes
‘I think I could always say that I could have planned something better with hindsight and this was no different, there were aspects which I had not considered. It would have been useful to spend perhaps a little more time beforehand reading up on the condition which I was investigating further’ (SSC4, 2011)
‘I sometimes should have asked for help sooner, rather than struggling to understand something on my own’ (SSC4, 2011)
‘I think I could have been more organised with my time at the beginning of the project. However it was only through trying to get over difficulties that I was able to find the best way to do things. If I had to do it again, I would have a much better idea of how to conduct the project’ (SSC4 2010)
‘Too easy to leave things [to the] last minute. Despite the [course organisers] best attempts to provide information I still felt unprepared for how much time some aspects of the project would take’ (SSC4, 2011)
**Have you any suggestions for changes to improve this SSC**
‘I feel that the marking for this SSC was unfair in that everybody got the same mark. I felt that within our group there were huge differences in the amount of work and effort that individuals put in and this is not reflected in the mark that people have received as well as making the peer feedback seem slightly irrelevant as there is no point in giving one person excellent feedback and another poor feedback if there is no way that this will influence their overall grades’ (SSC2a, 2010)

## International context of research in undergraduate medicine

An increased international acceptance of developing research skills during undergraduate training has emerged [17, 26, 27]. The Medical Education in Europe 2 [17] project has been established as part of the Bologna process to align degrees with common standards to facilitate the transfer of workforces throughout the European area. In initial studies in the mid-2000s, there was good consensus with all partners across Europe in defining most learning outcomes and competencies for a graduating doctor. However, at this time there was limited consensus in the importance of gaining competency in research skills. Less than a decade later, evidence-based medicine has grown and matured, and the validity of its practice more fully implemented. This has correspondingly required undergraduate medical curricula to respond, hence a range of basic research skills are now recognized as important learning outcomes [17].

## Conclusions

The SSC programme at the University of Edinburgh has some clear markers of success. Firstly, it delivers research opportunities to undergraduate medical students, and is important in defining what is different about the Edinburgh curriculum compared with many other UK medical schools. This is highlighted to potential applicants, with research publications arising with student authors presented as evidence. Secondly, it is now well established and has been shown to be sustainable and valued by Faculty, with hundreds involved as tutors and supervisors. Finally, student feedback indicates they clearly recognize and take the opportunities presented.

Medical students are the life-blood of a community of practice across clinical and academic medicine. They bring enthusiasm, and new ideas which may be naive or alternatively unbiased by existing dogma. Indeed, in the past students have made significant discoveries in medicine [43]. As developing researchers at this research-teaching nexus, they can contribute significantly to initiatives for establishing the evidence base, reviewing local practice through audit and service evaluation. Training at this stage builds research capacity to continue to contribute to the evidence base throughout their careers. Students also gain insight into biomedical and translational research, where there have been long stated concerns about how the future leaders in academic medicine are going to be found [44]. By involving and developing skills of all medical students in research, they will be better prepared for interpreting and engaging with change, uncertainty, risk and complexity to make informed decisions in their future medical practice.

The final comment should be from a year 4 student commenting on the best aspects of their project:

*Freedom to manage your own time; opportunity to focus in such a specific area; opportunity to work so closely as an individual [student] with a clinical specialist; an attachment where you genuinely get out as much as you put in.*

Essential practice points

- Research competencies are best developed through active experience and participation in learner-centred areas of the curriculum.
- Development of these research competencies will integrate well into a curriculum, to enable students to better engage with evidence-based medicine, and the complexity, risk and uncertainty of medical practice.
- Student-centred elements in the curriculum that offer choice, are effective at motivating and engaging students in their learning.
- Alignment of both faculty and student aims ensures sustainability and acceptability of curriculum initiatives to develop research competencies.
- Self-proposal of projects by students is effective at establishing good faculty engagement—trust your students!
- For assessment methods, look beyond the field of medical education at the teaching-research nexus in other subject areas.
